# Menke–Hennekam Syndrome: A Literature Review and a New Case Report

**DOI:** 10.3390/children9050759

**Published:** 2022-05-22

**Authors:** Aurora Sima, Roxana Elena Smădeanu, Anca Angela Simionescu, Florina Nedelea, Andreea-Maria Vlad, Cristina Becheanu

**Affiliations:** 1 Department of Pediatrics, Carol Davila University of Medicine and Pharmacy, “Grigore Alexandrescu” Emergency Hospital for Children, 011743 Bucharest, Romania; aurora.sima@umfcd.ro (A.S.); andreeamariavlad@yahoo.com (A.-M.V.); cristina.becheanu@umfcd.ro (C.B.); 2 Department of Obstetrics and Gynecology, Carol Davila University of Medicine and Pharmacy, Filantropia Clinical Hospital, 011132 Bucharest, Romania; anca.simionescu@umfcd.ro; 3 Department of Clinical Genetics, Carol Davila University of Medicine and Pharmacy, Filantropia Clinical Hospital, 011132 Bucharest, Romania; florina.nedelea@umfcd.ro

**Keywords:** Menke–Hennekam syndrome, whole exome sequence, exons 30 and 31, *CREBBP*, failure to thrive, developmental delay, cleft palate

## Abstract

Background: Menke–Hennekam syndrome (MHS) is a rare and recently described syndrome consecutive to the variants in exon 30 or 31 in *CREBBP* (CREB-binding protein gene). The CREB-binding protein (*CREBBP)* and *EP300* genes are two commonly expressed genes whose products possess acetyltransferase activity for histones and various other proteins. Mutations that affect these two genes are known to cause Rubinstein–Taybi syndrome (RTS); however, with the application of whole exome sequencing (WES) there were reports of variants that affect specific regions of exon 30 or 31 of these two genes but without the specific phenotype of RTS. Material and Methods: A review of the available literature was conducted, aimed at underscoring the difficulties in diagnosing MHS based on phenotype particularities. Results: Five applicable studies were identified by searching PubMed, Web of Science, and Scopus databases for publications up to November 2021 using the key terms “Menke–Hennekam syndrome” and “*CREBBP*”. Conclusions: In this paper, we present a new case and highlight the importance of exome sequencing to identify different mutations of exons 30 and 31 of the *CREBBP* gene involved in MHS, and we make formal recommendations based on our literature review.

## 1. Introduction

The CREB-binding protein (*CREBBP*) and *EP300* genes are ubiquitously expressed, functionally related genes whose products possess acetyltransferase activity for histones and various other proteins. Embryonic development and several cellular processes, such as growth and differentiation, are controlled by their activities [1]. Mutations that affect these two genes are recognized to cause Rubinstein–Taybi syndrome (RTS), and were detected in more than half of all patients diagnosed with this condition based on clinical features. Rubinstein–Taybi syndrome (RTS, MIM#180849) is a rare condition that was first reported in 1963. Although there are no specific criteria for the diagnosis, it comprises a series of distinctive constitutional features such as broad thumbs and halluces, beaked nose, low-set ears, arched palate, microcephaly, teeth anomalies, and an atypical “grimace-like” smile. Patients with RTS are also developmentally delayed. Genetic research identified mutations in the *CREBBP* and *EP300* genes as causative factors of this syndrome in up to 70% of patients [2]. Since 2016, with the application of whole-exome sequencing (WES), there were reports of variants that affect specific regions of exons 30 and 31 of these two genes but without the specific phenotype of RTS [3]. In 2016, Menke et al. reported on 11 cases of patients with mutations in certain *CREBBP* segments with phenotypic features that are distinct from those of RTS. Since then, several working groups described other individuals sharing the same genotype characteristics. In 2019, it was proposed that this be named the Menke–Hennekam syndrome (MHS) after the authors that first described it [3,4].

Menke–Hennekam syndrome (MHS) is a recently described de novo congenital disorder that may impose termination of pregnancy when it is prenatally diagnosed. Making an early diagnosis is important; it can improve future prospects and facilitate early intervention for various complications. Complex phenotypes require genetic counseling before and after the genetic test. Intellectual disabilities of varying degrees, impaired motor development, particular facial and other constitutional features, short stature, feeding difficulties, and autistic behavior are the most commonly reported characteristics. The genetic defects consist of mutations in exons 30 and 31 in the *CREBBP* gene, which are responsible for MHS type I (MIM#618332), and in the *EP300* gene, which are responsible for MHS type II (MIM#618333) [4,5]. Until 2016, it was known that the mutations in these two genes generated RTS or that they were associated with different types of cancer [6].

Products of the *CREBBP* and *EP300* genes are widely distributed in human tissues and involved in embryonic development and important biological processes such as cell growth and specific cell function development (differentiation). They have acetyltransferase activity on histones and other proteins, we agree with the strikethrough and participate as co-activators in regulating transcription. Given their roles in critical biological processes, it is reasonable to believe that any alteration in their expression can lead to complex developmental anomalies [6,7,8,9]. The *CREBBP* locus was identified on chromosome 16p13.3, and is homologous with the *EP300* gene on chromosome 22q13.2 [7,9]. The gene-encoding protein CREB has a modular structure and consists of several domains, including three zinc finger domains. The p300 protein is highly homologous to CREB-binding protein, with a 70% shared identity in structural amino acids [10,11]. The ZNF2 and ZNF3 zinc domains are involved in zinc binding and represent the site of different variants in exons 30 and 31 of the *CREBBP* gene [11].

## 2. Materials and Methods

A systematic literature search of the PubMed, Web of Science, and Scopus databases was conducted. Full-length articles published in peer-reviewed journals up to November 2021 were selected. The keywords used in the search strategy were “Menke–Hennekam syndrome” and “*CREBBP*”. The study was conducted in accordance with the Declaration of Helsinki, and the publication of this case study was approved by the Hospital’s Ethics Committee (31289).

## 3. Results

Using the strategy outlined in Section 2, seven articles were identified [3,4,5,11,12,13,14]. We selected only full-text articles (*n* = 5) and excluded meeting abstracts (*n* = 2). Complete data on genotype are presented in Table 1.

There were 32 individuals with MHS type I and only 2 individuals with MHS type II described in the literature. In 31 individuals with MHS type I, the mutations were de novo; inheritance for 1 individual could not be established (Table 1) [5,11].

The phenotypes are similar in both types of MHS (Table 2), and may include the following features: facial dysmorphism (narrow eyelid slits, flattened nose base, short nose, anteverted nostrils, short philtrum, and telecanthus), hearing or vision disorders, finger malformations, recurrent infections of the upper respiratory tract, eating disorders with low weight and short stature, microcephaly, mental retardation of varying degrees, epilepsy, deafness, autism spectrum disorders, and other behavioral disorders. Rarely, there are malformations such as cleft palate, congenital heart malformations, renal malformations, malrotations, cryptorchidism, and cerebral malformations (white matter atrophy and agenesis of the corpus callosum) [11]. The most severe conditions are neuromotor disabilities and eating disorders [4]. Diagnosis is based on the existence of a clinical suspicion due to the presence of a characteristic phenotype, and confirmation involves sequencing the *CREBBP* and *EP300* genes using NGS [3].

There is no specific treatment for this condition. The management of MHS cases requires a multidisciplinary team consisting of a maternal-fetal medicine specialist, pediatrician, geneticist, pediatric surgeon, pediatric neurologist, ophthalmologist, ENT specialist, nutritionist, and psychologist. The prognosis of this condition is not known, and the evolution is dictated by the associated malformations and potential complications.

## 4. Case Report

**Figure 1:**
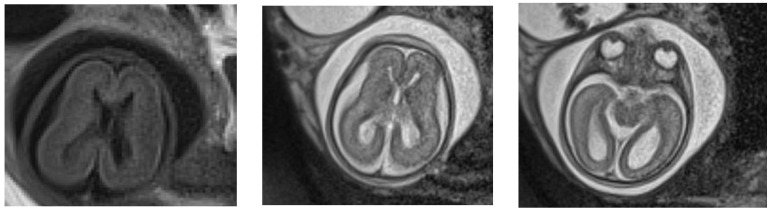
MRI at 24 weeks of gestation: no Sylvian fissure are seen; ventricular dilatation; the parieto-occipital and Sylvian fissures appear flat and the subarachnoid space is increased.

We present the case of a two-month-old male infant from a mixed family (his mother is Romanian and his father is Syrian) admitted to the Pediatrics Department of the “Grigore Alexandrescu” Emergency Hospital for Children in Bucharest for feeding difficulties and growth failure. Intrauterine growth restriction, oligohydramnios, and ventriculomegaly were diagnosed during pregnancy. The mother was monitored for intrauterine growth restriction and oligohydramnios; ventriculomegaly was reported in the second trimester of pregnancy. In the first trimester, combined screening for aneuploidy and preeclampsia indicated a low risk of their development. A fetal MRI was performed, and there was also suspicion of a cleft palate (Figure 1).The parents decided to continue the pregnancy. At 38 weeks of pregnancy, the infant was born via cesarean section due to intrauterine growth restriction. The birth weight was 2260 g (−2.4 SD, <p1%), the length was 43 cm (−3.5 SD, <p1%), and the head circumference was 30 cm (−3.5 SD, <p1%); the Apgar scores were nine at 1 minute and ten at 5 minutes. After birth, the clinical examination revealed dysmorphic facial features (hypertelorism, microphthalmia, mongoloid palpebral fissures, microretrognathia, and long philtrum), membranous partial cleft palate, low-set ears, and prominent occiput. The infant also had an umbilical and inguinal hernia, syndactyly of the third and fourth digits of the feet, fifth digit clinodactyly, floating testicles, axial hypotonia, and limb hypertonia, which raised clinical suspicion regarding a genetic syndrome.

The infant was fed with a special milk formula for premature infants since birth; however, the daily intake was poor (only 300 mL). Consequently, his weight gain was below normal (600 g in six weeks).

**Figure 2:**
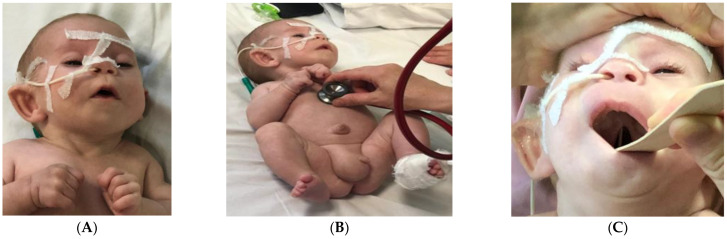
Phenotypical characteristics: facial dysmorphism (**A**), limb hypertension, malformations of the fingers, umbilical and inguinoscrotal hernias (**B**), and partial cleft palate (**C**).

In May 2019, at the age of two months, the infant was admitted to the Pediatrics Department of the “Grigore Alexandrescu” Emergency Hospital for Children in Bucharest for feeding difficulties and growth failure. Clinical examination at admission revealed a dystrophic infant (weight 2710 g, Z score −3.12) with facial dysmorphism (hypertelorism, microphthalmia, eyelid ptosis, mongoloid slant and bilateral blepharophimosis, anteverted nostrils, long philtrum, micrognathia, and low-set dysplastic posteriorly rotated ears) and other constitutional anomalies (presacral fossa, cleft palate, clinodactyly of the fifth finger of both hands, abnormally implanted toes, and syndactyly of the third and fourth toes of the left foot). Umbilical and right inguinoscrotal hernias were also observed (Figure 2 A–C).

Neurologic examination indicated global developmental delay. A cerebral CT scan was conducted and revealed asymmetric lateral ventricles. The cardiological evaluation revealed an atrial septal defect with a small left-to-right shunt without hemodynamic impact. Ophthalmologic examination established the diagnosis of severe bilateral eyelid ptosis that required surgical treatment and hypermetropia that required postoperative optical correction. As indicated by the clinical geneticist, blood samples for microarray testing were obtained. The results were normal, without any significant copy number variations identified.

Initially, tube feeding was recommended, with prior bottle feeding to stimulate the swallowing reflex. The patient was discharged with a nasogastric tube and followed a continuous physical therapy program.

**Figure 3:**
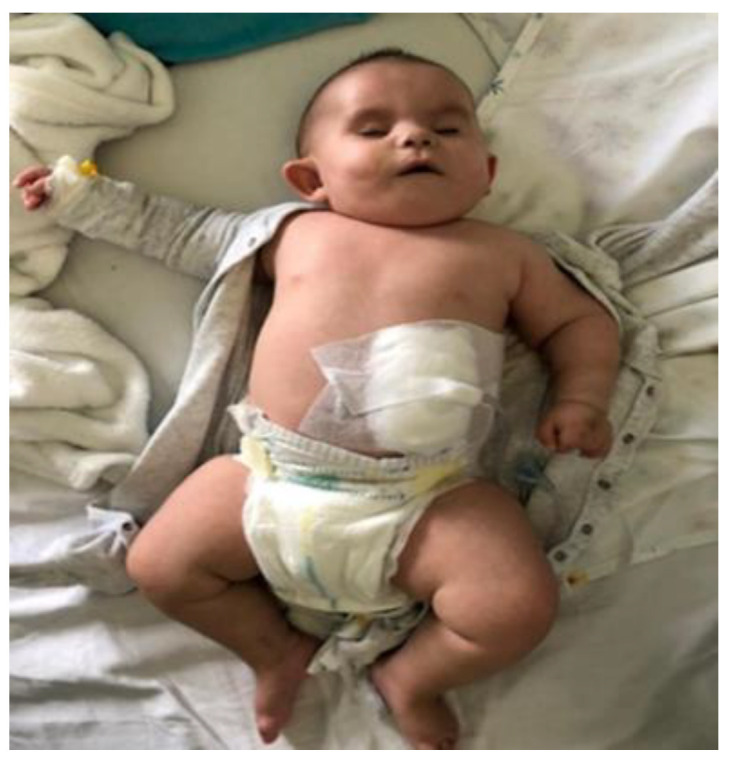
Clinical aspects at the age of seven months.

At the age of seven months the infant required gastrostomy as a result of persistent feeding difficulties with a low intake and consequent failure to thrive (Figure 3).

Considering the normal microarray results and the possible heterogenous etiology of this complex phenotype, WES was performed, which identified the pathogenic variant c.5602C > T (p.Arg1868Trp) in the *CREBBP* gene, confirming a diagnosis of MHS type I. In the vast majority of previously published cases, as in our case, the mutation is *de novo*. Therefore, the risk of recurrence is statistically low. Keeping in mind the severity of the phenotype and the possibility of germinal mosaicism, prenatal diagnosis was advised for future pregnancies.

The subsequent disease evolution was marked by persistent developmental delay, severe hypotonia, and frequent hospital admissions. The patient presented recurrent vomiting and one episode of aspiration pneumonia. Corrective surgery for cleft palate was performed at eighteen months (one procedure) and for eyelid ptosis at two years. At eleven months, he was diagnosed with bilateral neurosensorial hypoacusis, for which bilateral cochlear implants were inserted. The atrial septal defect closed spontaneously before the age of seven months.

At the last check-up, at twenty-four months, the patient was sitting unsupported; he was vocalizing but had not achieved his first-word utterance, and was still fed through a gastrostomy.

At the time of writing, the family had a second healthy child. During pregnancy, prenatal diagnosis via testing amniotic fluid for chromosomal anomalies (microarray) and Sanger sequencing for the previous variant, c.5602C > T (p.Arg1868Trp) of the *CREBBP* gene was undertaken; postnatal evaluation revealed normal results.

## 5. Discussion

This case presents the importance of WES in the diagnosis of rare syndromes, including MHS. It is worth mentioning that in a practical clinical setting, after the identification of a plurimalformative and dysmorphic newborn, the investigation protocol may begin with a microarray test, eventually followed by a multigene panel for neurodevelopmental delay, or, ideally, WES analysis. According to Banka et al., the data for the 32 patients currently reported (November 2021) support the existence of an “exon 30/31 *CREBBP* missense variant syndrome” in individuals without a specific RTS phenotype [4]. In 31 cases, the identified mutations were de novo. In one case, inheritance could not be established (patient 16, Menke et al., 2018) [5,11]. The previously described 32 patients share some common features such as an intellectual disability of variable severity, and in most cases, similar facial features, short stature, and microcephaly; however, there are other phenotypic characteristics. Several patients have behavioral issues resembling autism, and feeding problems that often require gastrostomy. Malformations such as cleft palate, cardiac anomalies, cryptorchidism, and finger anomalies (syndactyly, sandal gap, and clinodactyly) are infrequently observed. Brain anomalies such as corpus callosum agenesis/hypoplasia, enlarged ventricles, and cerebral atrophy were reported [11,12].

So far, there are only six individuals described with the same *CREBBP* variant as found in our patient, c.5602C > T (i.e., patient 2 of the Banka et al. report, patients 9 and 10 of the 2016 Menke et al. report, and patients 17–19 of the 2018 Menke et al. report) (Table 3) [3,4,11]. These patients share some common features such as severe neurological and motor impairments, microcephaly, mongoloid slant, eyelid ptosis, short nose, long philtrum, low-set ears, and impaired hearing that, in some cases, required cochlear implants. Often, these patients are not able to walk and have no speech development. All of these patients had feeding issues, and half required gastrostomy (Table 2) [3,4,11].

The particular phenotype of these seven patients may constitute, by its severity, a distinct subgroup in patients with MHS, as suggested by Banka et al. [4]. Furthermore, the observations of its clinical homogeneity may be a foundation for establishing genotype–phenotype correlations in this pleomorphic condition.

This case report underscores the importance of genetic testing in individuals with severe neurological impairments and feeding problems in association with other particular constitutional features that may resemble a syndromic condition. Reporting such cases is mandatory in order to collect data on the genotype and phenotype of these individuals for more accurate classification. Given the phenotype heterogeneity, it is reasonable to think that with each new reported individual diagnosed with MHS, we are closer to a more precise characterization of this syndrome.

## 6. Conclusions

Considering that it is a recently described condition, MHS can cause major problems for diagnosis and treatment, including prenatal counseling. Indeed, there were many cases of MHS before 2016; until then, they were undiagnosed. The progress of medical genetics allows us to define this syndrome and to successfully diagnose children with MHS. Early diagnosis allows us to inform parents about prognosis and provide closer follow-up, with the possibility of preventing or quickly diagnosing possible complications. Currently, research on this topic is limited; however, we expect that protocols for monitoring and treatment of these cases will be developed over time as more information is collected.

## Figures and Tables

**Table 1:** The genotype of individuals reported with *CREBBP*/*EP300* variants.

Patient	Sex	Gene	Variant	Inheritance	Studies
M1	M	*CREBBP*	c.5128T > C (p.Cys1710Arg)	de novo	Menke et al., 2016 [3]
M2	M	*CREBBP*	c.5240T > G (p.Leu1747Arg)	de novo	
M3	M	*CREBBP*	c.5357G > C (p.Arg1786Pro)	de novo	
M4	M	*CREBBP*	c.5456G > T (p.Cys1819Phe)	de novo	
M5	M	*CREBBP*	c.5478C > G (p.Cys1826Trp)	de novo	
M6	F	*CREBBP*	c.5513G > A (p.Cys1838Tyr)	de novo	
M7	M	*CREBBP*	c.5599C > T (p.Arg1867Trp)	de novo	
M8	F	*CREBBP*	c.5600G > A (p.Arg1867Gln)	de novo	
M9	F	*CREBBP*	c.5602C > T (p.Arg1868Trp)	de novo	
M10	F	*CREBBP*	c.5602C > T (p.Arg1868Trp)	de novo	
M11	F	*CREBBP*	c.5614A > G (p.Met1872Val)	de novo	
M12	M	*CREBBP*	c.5155C > G (p.His1719Asp)	de novo	Menke et al., 2018 [11]
M13	M	*CREBBP*	c.5345C > T (p.Ala1782Val)	de novo	
M14	F	*CREBBP*	c.5485C > G (p.His1829Asp)	de novo	
M15	F	*CREBBP*	c.5595_5597del(p.Met1865_Arg1866delinslle)	de novo	
M16	M	*CREBBP*	c.5600G > A (p.Arg1867Gln)	unknown	
M17	M	*CREBBP*	c.5602C > T (p.Arg1868Trp)	de novo	
M18	M	*CREBBP*	c.5602C > T (p.Arg1868Trp)	de novo	
M19	F	*CREBBP*	c.5602C > T (p.Arg1868Trp)	de novo	
M20	M	*CREBBP*	c.5603G > A (p.Arg1868Gln)	de novo	
M21	F	*CREBBP*	c.5608G > C (p.Ala1870Pro)	de novo	
M22	M	*CREBBP*	c.5614A > G (p.Met1872Val)	de novo	
E 1	F	*EP300*	c.5471A > C (p.Gln1824Pro)	de novo	
E 2	F	*EP300*	c.5492_5494del (p.Arg181del)	de novo	
A1	M	*CREBBP*	c.5170G > A (p.Glu1724Lys)	de novo	Angius et al., 2019 [12]
B1	M	*CREBBP*	c.5357G > A (p.Arg1786His)	de novo	Banka et al., 2019 [4]
B2	F	*CREBBP*	c.5602C > T (p.Arg1868Trp)	de novo	
B3	F	*CREBBP*	c.5354G > A (p.Cys1785Try)	de novo	
N1	F	*CREBBP*	c.5570_5590del	de novo	Nishi et al., 2021 [5]
N2	M	*CREBBP*	c.5614A > G (p.Met1872Val)	de novo	
N3	M	*CREBBP*	c.5614A > G (p.Met1872Val)	de novo	
N4	M	*CREBBP*	c.5991delC (p.Val1998)	de novo	
N5	M	*CREBBP*	c.6188C > G (p.Ser2063)	de novo	
N6	F	*CREBBP*	c.6241C > T (p.Gln2081Ter)	de novo	

**Table 2:** The incidence of several typical features of MHS.

Feature	Menke et al. 2018 (*n* = 24, %) [11]	Banka et al. 2019 (*n* = 3, %) [4]	Angius et al. 2019 (*n* = 1, %) [12]	Nishi et al. 2021 (*n* = 6, %) [5]
Intrauterine growth restriction	7 (29)	2 (67)	-	5 (84)
Microcephaly	10/23 (43)	3 (100)	-	5 (84)
Downslant (D)/upslant (U) palpebral fissures	3D, 14U (13, 58)	1D (34)	n.a.	6U (100)
Epicanthus/telecanthus	13T, 5E (54, 21)	-	1E (100)	6E (100)
Philtrum long (L)/short (S)/deep (D)	4S, 12L, 6D (17, 50, 25)	1L/D (34)	-	6L (100)
Low-set ears	12 (50)	2 (67)	1 (100)	4 (67)
Ptosis (P)/blepharophimosis (B)	8P, 10B (33, 42)	1P (34)	-	1P (17)
Hypertelorism	n.a.	1 (34)	1 (100)	6 (100)
Depressed nasal bridge	13 (54)	n.a.	1 (100)	n.a.
Short nose	12 (50)	2 (67)	-	4 (67)
Clinodactyly	6 (25)	-	1 (100)	3 (50)
Cardiac involvement	4 (17)	1 (34)	-	1 (17)
Intellectual impairment	19/21–24 (80–90)	3 (100)	1 (100)	6 (100)
Autistic-like behaviour	13/20–24 (54–65)	1 (34)	-	1 (17)

**Table 3:** Clinical features of the seven patients with the same *CREBBP* gene variant (c.5602C > T).

	M9	M10	B2	M17	M18	M19	OUR PATIENT
Age at diagnosis (years)	4	0.8	0.7	2	4	1	0.4
Gender	F	F	F	M	M	F	M
Prenatal growth retardation	−	+	+	+	+	+	+
Microcephaly	+	+	+	n.a	−	−	+
Highly arched eyebrows	+	−	−	n.a	n.a	n.a	+
Palpebral fissures upslanted (U)/downslanted (D)	U	U	n.a	U	−	U	U
Ptosis(P)/blepharophimosis (B)	P	P	−	P/B	B	P/B	P/B
Telecanthus	+	+	n.a	+	+	+	n.a
Low set ears	+	+	+	+	+	+	+
Short nose	+	+	+	+	+	+	+
Long philtrum	+	+	+	+	−	+	+
Thin vermilion of upper lip	−	−	n.a	−	+	+	+
Cleft palate	n.a	n.a	n.a	−	+	−	+
High palate	+	−	n.a	+	+	+	−
Severe intellectual disability	+	n.a	+	+	+	n.a	+
Cardiac anomalies	−	−	−	−	−	−	atrial septal defect
Scoliosis	−	−	+	−	+	+/−	−
Age at walking	7 yr	−	−	−	4 yr	n.a	−
Age at first words	−	−	−	−	−	−	−
Syndactyly	+	−	−	+	−	−	+
Feeding disorders/gastrostomy (G)	+/G	+	+/G	−	+/G	+	+ (G)
Hypoacusis	+	+	+	+/−	+	−	+
